# Exploiting mitochondrial targeting signal(s), TPP and bis-TPP, for eradicating cancer stem cells (CSCs)

**DOI:** 10.18632/aging.101384

**Published:** 2018-02-19

**Authors:** Bela Ozsvari, Federica Sotgia, Michael P. Lisanti

**Affiliations:** 1 Translational Medicine, School of Environment and Life Sciences, Biomedical Research Centre (BRC), University of Salford, Greater Manchester, United Kingdom; 2 The Paterson Institute, University of Manchester, Withington, United Kingdom

**Keywords:** tri-phenyl-phosphonium (TPP), cancer stem cells (CSCs), cancer therapy, mitochondrial targeting signal

## Abstract

Tri-phenyl-phosphonium (TPP) is a non-toxic chemical moiety that functionally behaves as a mitochondrial targeting signal (MTS) in living cells. Here, we explored the hypothesis that TPP-related compounds could be utilized to inhibit mitochondria in cancer stem cells (CSCs). We randomly selected 9 TPP-related compounds for screening, using an ATP depletion assay. Based on this approach, five compounds were identified as “positive hits”; two had no detectable effect on ATP production. Remarkably, this represents a >50% hit rate. We validated that the five positive hit compounds all inhibited oxygen consumption rates (OCR), using the Seahorse XFe96 metabolic flux analyzer. Interestingly, these TPP-related compounds were non-toxic and had little or no effect on ATP production in normal human fibroblasts, but selectively targeted adherent “bulk” cancer cells. Finally, these positive hit compounds also inhibited the propagation of CSCs in suspension, as measured functionally using the 3D mammosphere assay. Therefore, these TPP-related compounds successfully inhibited anchorage-independent growth, which is normally associated with a metastatic phenotype. Interestingly, the most effective molecule that we identified contained two TPP moieties (i.e., bis-TPP). More specifically, 2-butene-1,4-bis-TPP potently and selectively inhibited CSC propagation, with an IC-50 < 500 nM. Thus, we conclude that the use of bis-TPP, a “dimeric” mitochondrial targeting signal, may be a promising new approach for the chemical eradication of CSCs. Future studies on the efficacy of 2-butene-1,4-bis-TPP and its derivatives are warranted. In summary, we show that TPP-related compounds provide a novel chemical strategy for effectively killing both i) “bulk” cancer cells and ii) CSCs, while specifically minimizing or avoiding off-target side-effects in normal cells. These results provide the necessary evidence that “normal” mitochondria and “malignant” mitochondria are truly biochemically distinct, removing a significant barrier to therapeutically targeting cancer metabolism.

## Introduction

Cancer stem cells (CSCs) are tumor-initiating cells (TICs) [1,2] that appear to be the biological basis of treatment failure, due to tumor recurrence and distant metastasis [3–7], ultimately leading to poor clinical outcome in cancer patients. As a consequence, new therapies are urgently needed, to specifically target and eradicate CSCs [1–7]. Circulating tumor cells (CTCs) also directly share many key functional properties with CSCs [1–7].

Interestingly, recent studies indicate that one unique feature of CSCs is a characteristic increase in mitochondrial mass [8], which may reflect a more strict dependence on mitochondrial function or OXPHOS [9,10]. Several independent lines of evidence support the idea that increased mitochondrial biogenesis or higher levels of mitochondrial protein translation may occur in CSCs [8–18]. For example, unbiased proteomics analysis directly shows that mitochondrial mass is elevated in CSCs [8].

Moreover, MitoTracker (a fluorescent mitochondrial dye) can be used successfully as a marker to identify and purify CSCs [9,10]. Specifically, the “Mito-high” cell population showed the greatest capacity for increased i) anchorage-independent growth and ii) tumor-initiating ability *in vivo* [10].

High telomerase activity also directly correlated with high mitochondrial mass and the ability of CSCs to undergo proliferative expansion [13]. Similarly, high mitochondrial mass in CSCs was also specifically associated with mitochondrial ROS production (hydrogen peroxide) [12] and could be targeted with either: i) mitochondrial anti-oxidants [19,20], ii) inhibitors of mitochondrial biogenesis (doxycycline) [11,13,14,18] or OXPHOS (atovaquone) [11,16,17], and even iii) inhibitors of cell proliferation (palbociclib, a CDK4/6 inhibitor) [13].

The above findings immediately suggest a new approach for the eradication of CSCs, via the development of novel mitochondrial inhibitors. Here, we provide a new strategy for the identification of novel non-toxic mitochondrial inhibitors, using TPP-based compounds, with proof-of-concept.

TPP serves as a chemical mitochondrial targeting signal [21]. Importantly, we directly showed that these TPP compounds are non-toxic in normal fibroblasts, but potently target CSC propagation, with an IC-50 as low as 500 nM. Using this approach, 2-butene-1,4-bis-TPP was the most effective compound that we identified.

## RESULTS

### Selective targeting of cancer cell mitochondria with TPP compounds

In order to identify new molecules that can be used to target mitochondria within CSCs, we devised a screening approach, by employing CellTiter-Glo to measure intracellular levels of ATP in adherent cancer cells (MCF-7). As ~85% of cellular ATP is normally derived from mitochondrial metabolism, ATP levels are an excellent read-out to monitor mitochondrial function. In parallel, the same 96-well plates were also stained with Hoechst 33342, to measure DNA content, to allow us to gauge cell viability.

**Figure 1:**
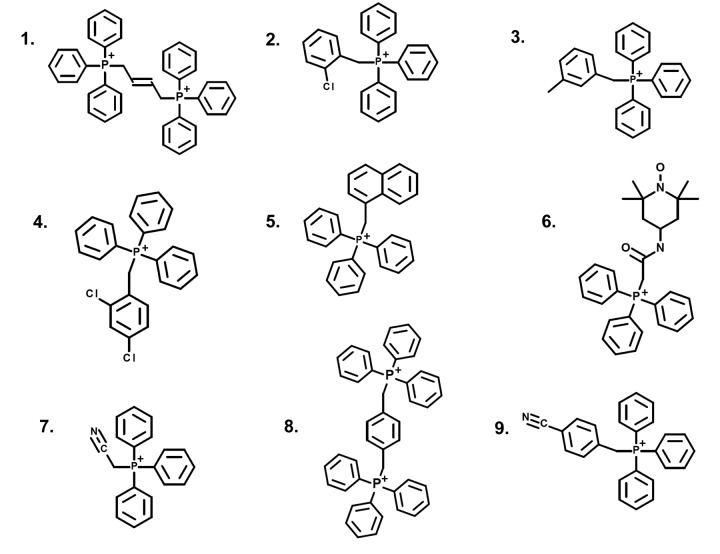
**Structures of nine TPP derivatives.** (1) 2-butene-1,4-bis-TPP; (2) 2-chlorobenzyl-TPP; (3) 3-methylbenzyl-TPP; (4) 2,4-dichlorobenzyl-TPP; (5) 1-naphthylmethyl-TPP; (6) mito-TEMPO; (7) cyanomethyl-TPP; (8) p-xylylene-bis-TPP; (9) 4-cyanobenzyl-TPP.

Tri-phenyl-phosphonium (TPP) is a well-established chemical mitochondrial targeting signal. Cargo molecules covalently attached to TPP accumulate within the mitochondria of living cells. Therefore, we randomly selected 9 TPP derivatives and subjected them to screening in our assay system. The chemical structures of these TPP-derivatives are shown in Figure 1.

Notably, five out of the nine TPP compounds were “positive hits” that significantly reduced ATP levels. These positive hits included: 2-butene-1,4-bis-TPP, 2-chlorobenzyl-TPP, 3-methylbenzyl-TPP, 2,4-dichlorobenzyl-TPP and 1-naphthylmethyl-TPP. This represents a hit rate of >50%.

However, two compounds were completely ineffective in reducing ATP levels (See Table 1). This finding is consistent with previous studies showing that the TPP moiety is not intrinsically toxic for normal cell mitochondria [21].

**Table 1:** Four ineffective TPP compounds.

	**Hoechst staining (%)**	**ATP level (%)**
mito-TEMPO*	**100.0**	**100.0**
**cyanomethyl-TPP**	**97.2**	**95.1**
**4-cyanobenzyl-TPP**	**72.7**	**68.2**
**p-xylylene-bis-TPP**	**69.1**	**43.9**

*(2‐(2,2,6,6‐tetramethylpiperidin‐1‐oxyl‐4‐ylamino)‐2‐oxoethyl‐TPP. Cell viability and intracellular ATP levels were determined in the same treated samples. MCF‐7 cell line, 50 μM treatment for 72 hours.

**Figure 2:**
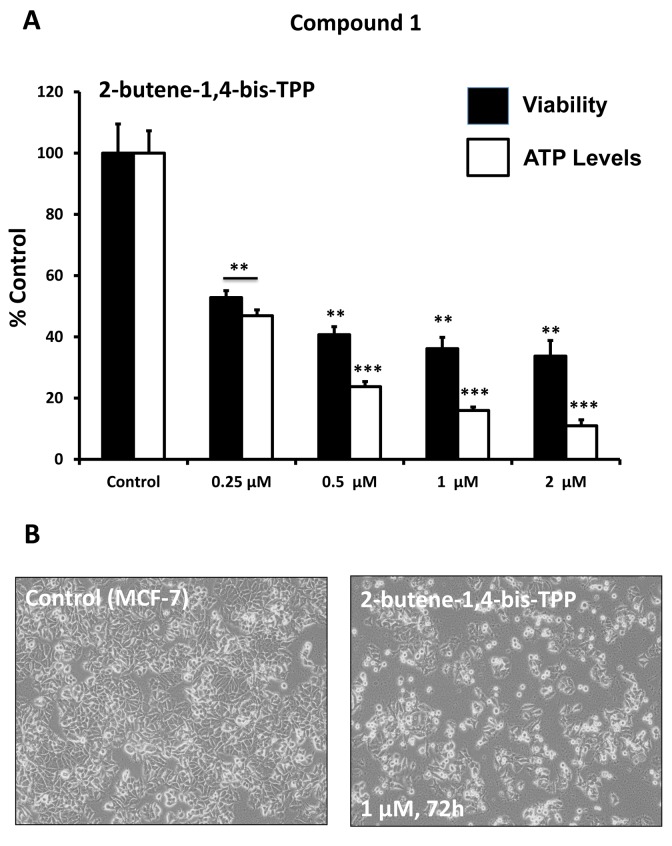
**Effect of TPP derivatives on cell viability and intracellular ATP levels in MCF-7 human breast cancer cells: Compound 1.** Cell viability and intracellular ATP levels were determined in the same treated samples. Hoechst staining (%) (shown in black bars); ATP levels (%) indicated in white bars. MCF-7 cells were treated for 72h. Data are represented as mean +/- SEM. Note that 2-butene-1,4-bis-TPP depletes ATP levels, relative to cell number. **p < 0.01; ***p < 0.001; indicates significance, all relative to the control.

**Figure 3:**
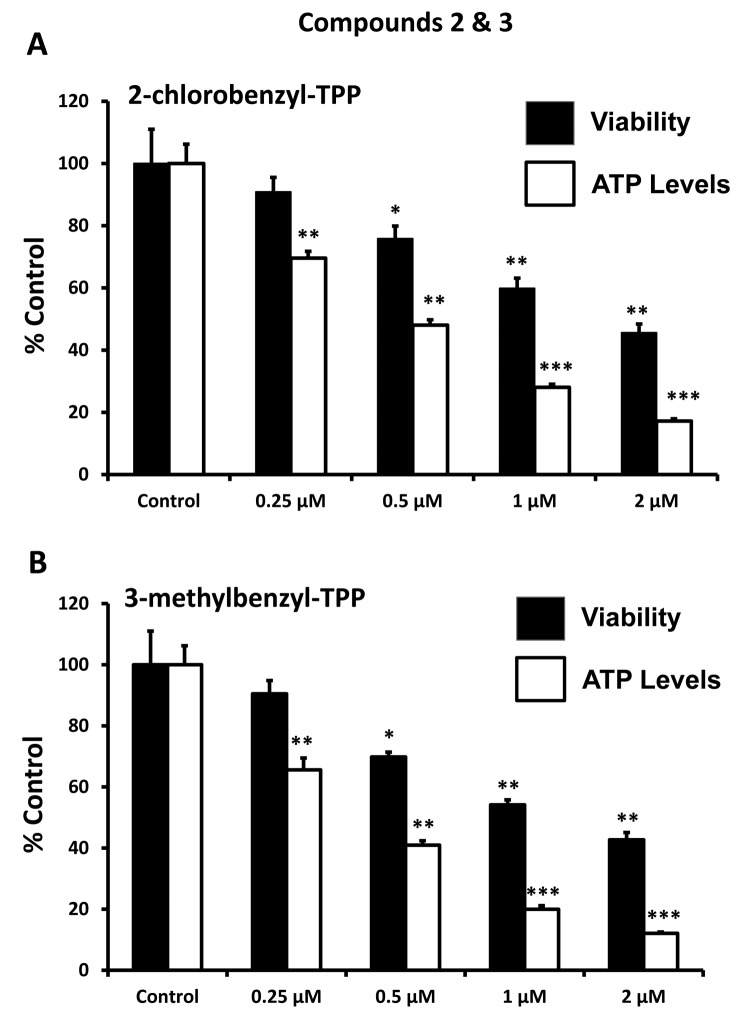
**Effect of TPP derivatives on cell viability and intracellular ATP levels in MCF-7 human breast cancer cells: Compounds 2 and 3.** Cell viability and intracellular ATP levels were determined in the same treated samples. Hoechst staining (%) (shown in black bars); ATP levels (%) indicated in white bars. MCF-7 cells were treated for 72h. Data are represented as mean +/- SEM. Note that both 2-chlorobenzyl-TPP and 3-methylbenzyl-TPP progressively deplete cellular ATP levels. *p < 0.05; **p < 0.01; ***p < 0.001; indicates significance, all relative to the control.

**Figure 4:**
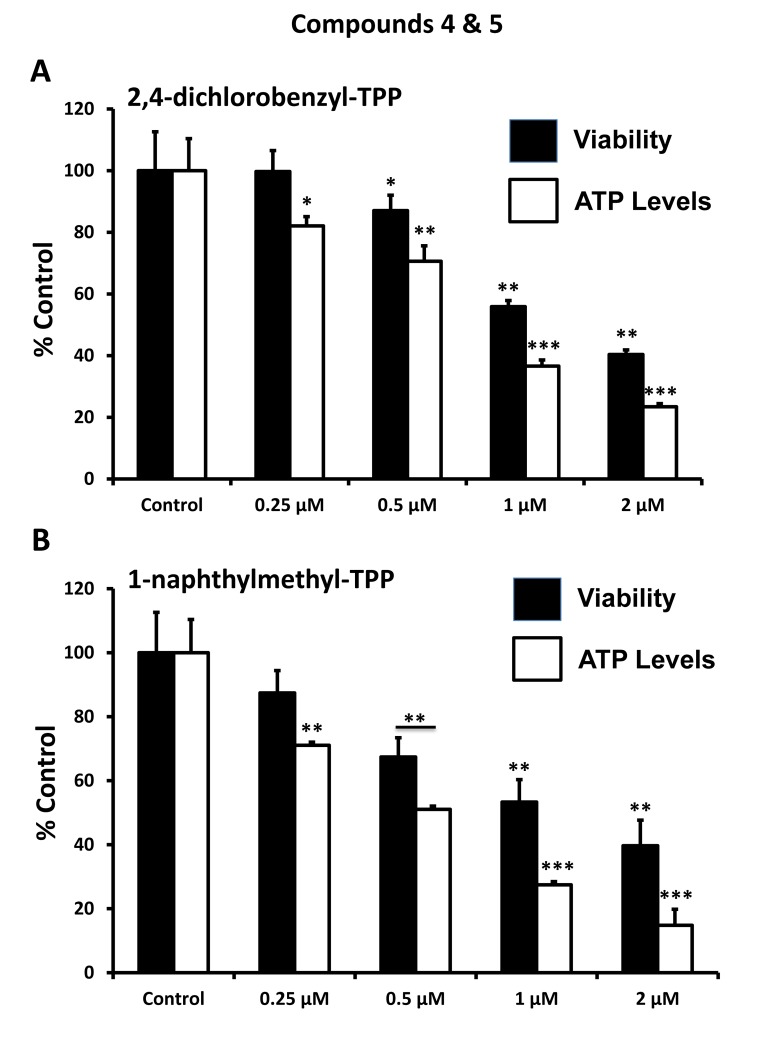
**Effect of TPP derivatives on cell viability and intracellular ATP levels in MCF-7 human breast cancer cells: Compounds 4 and 5.** Cell viability and intracellular ATP levels were determined in the same treated samples. Hoechst staining (%) (shown in black bars); ATP levels (%) indicated in white bars. MCF-7 cells were treated for 72h. Data are represented as mean +/- SEM. Note that both 2,4-dichlorobenzyl-TPP and 1-naphtylmethyl-TPP progressively deplete cellular ATP levels. *p < 0.05; **p < 0.01; ***p < 0.001; indicates significance, all relative to the control.

After initial screening, the five positive hit compounds were then subjected to further validation studies, shown in Figures 2-4, demonstrating that these TPP compounds are highly active in the range of 0.5 to 2 μM. Based on this initial analysis, 2-butene-1,4-bis-TPP appeared to be the most potent.

**Figure 5:**
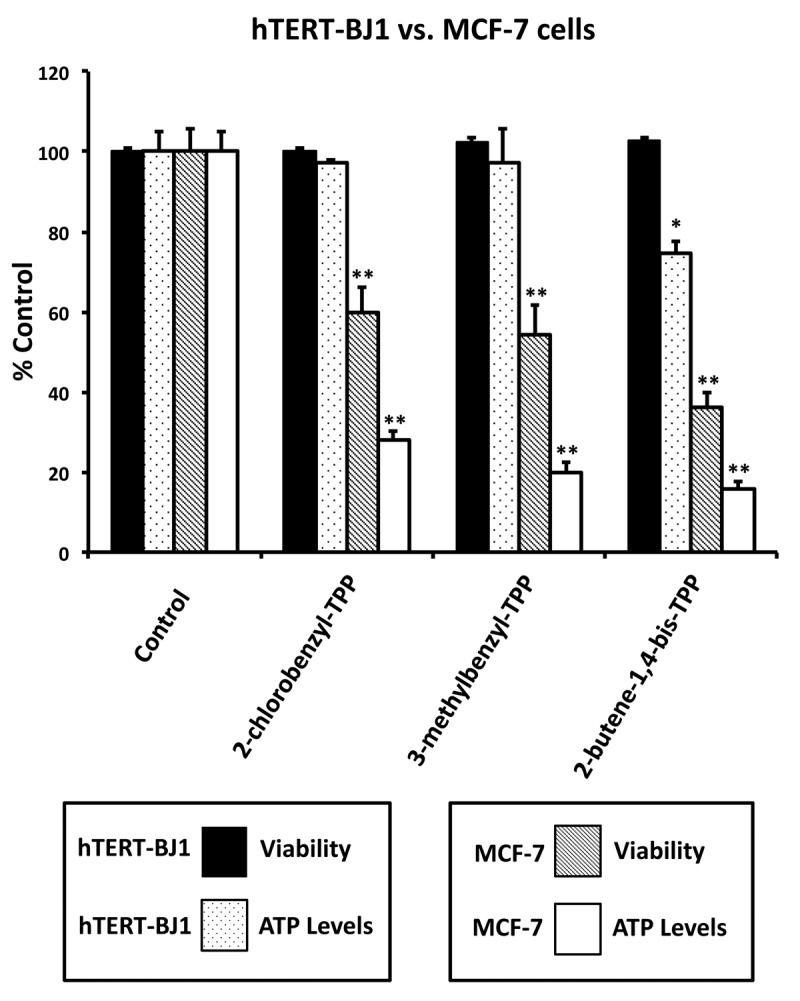
**Effects of TPP derivatives on cell viability and intracellular ATP levels in normal fibroblasts (hTERT-BJ1) and human breast cancer cells (MCF-7).** Cell viability and intracellular ATP levels were determined in the same treated samples. Hoechst staining (%) of hTERT-BJ1 human fibroblasts (black); ATP level (%) of hTERT-BJ1 human fibroblasts (dotted); Hoechst staining (%) of MCF-7 cells (inclined lines); ATP level (%) of of MCF-7 cells (white). TPP treatments at 1 µM, 72h. Data are represented as mean +/- SEM. *p < 0.05; **p < 0.01; indicates significance, all relative to the control.

Figure 5 highlights that these TPP compounds are relatively non-toxic in normal human fibroblasts (hTERT-BJ1), but are preferentially active in cancer cells (MCF-7). For example, in human fibroblasts, 2-butene-1,4-bis-TPP had no effect on cell viability and only mildly reduced ATP levels by 25%. In contrast, at the same concentration (1 μM) in MCF-7 cancer cells, 2-butene-1,4-bis-TPP reduced cell viability by nearly 65% and decreased ATP levels by almost 85%. Therefore, 2-butene-1,4-bis-TPP was 2.8-fold more effective at reducing cell viability in cancer cells (versus fibroblasts). Similarly, 2-butene-1,4-bis-TPP was 4.7-fold more effective at reducing ATP levels in cancer cells, relative to normal fibroblasts. Very similar results were obtained with the other TPP compounds that we evaluated.

### Identifying TPP compounds that target “malignant” mitochondria in CSCs

**Figure 6:**
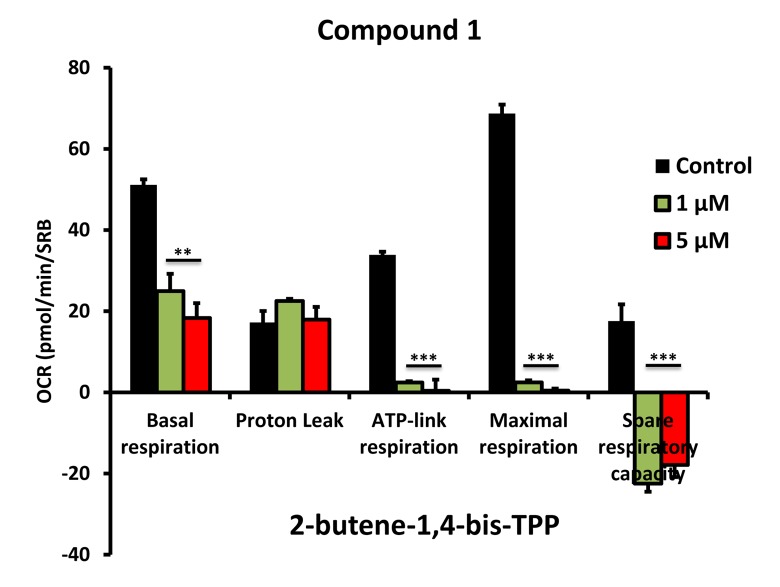
**Impaired mitochondrial function of MCF-7 cells after treatment with various TPP derivatives: Compound 1.** Oxygen consumption rate (OCR) was measured with a Seahorse XF96 Extracellular Flux Analyzer. Data are represented as mean +/- SEM. Note that 2-butene-1,4-bis-TPP effectively inhibits mitochondrial oxygen consumption. **p < 0.01; ***p < 0.001; indicates significance, all relative to the control.

**Figure 7:**
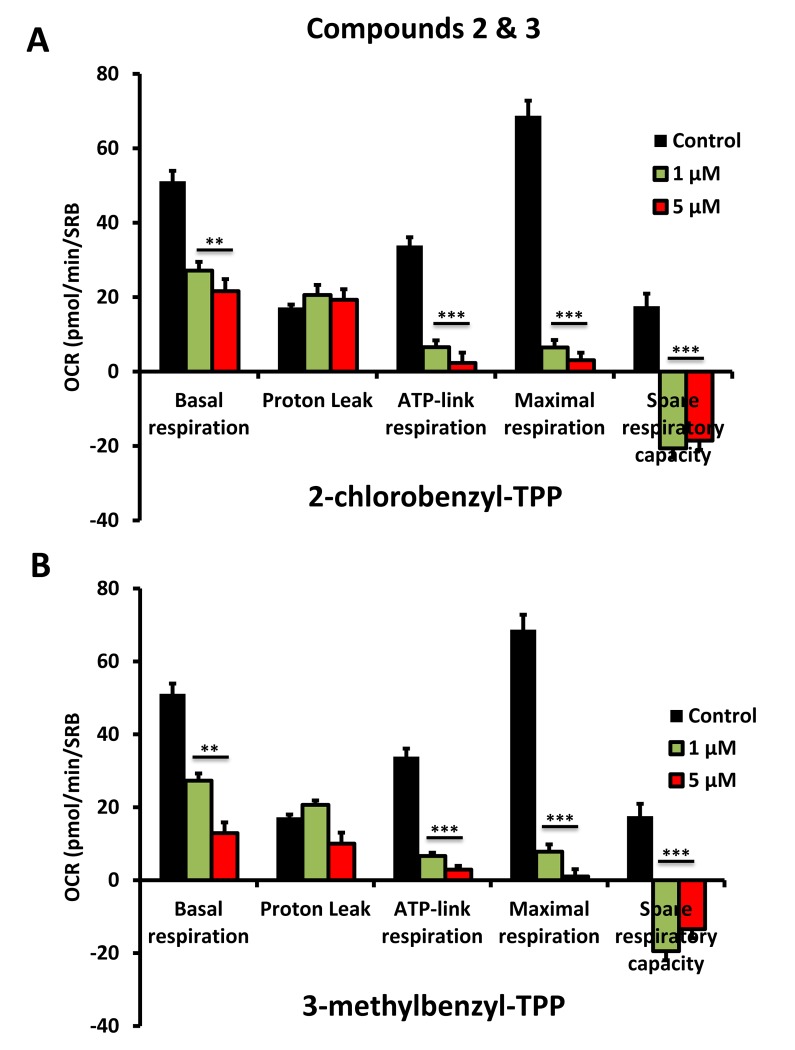
**Impaired mitochondrial function of MCF-7 cells after treatment with various TPP derivatives: Compounds 2 and 3.** Oxygen consumption rate (OCR) was measured with a Seahorse XF96 Extracellular Flux Analyzer. Data are represented as mean +/- SEM. Note that 2-chlorobenzyl-TPP and 3-methylbenzyl-TPP both effectively inhibit mitochondrial oxygen consumption. **p < 0.01; ***p < 0.001; indicates significance, all relative to the control.

**Figure 8:**
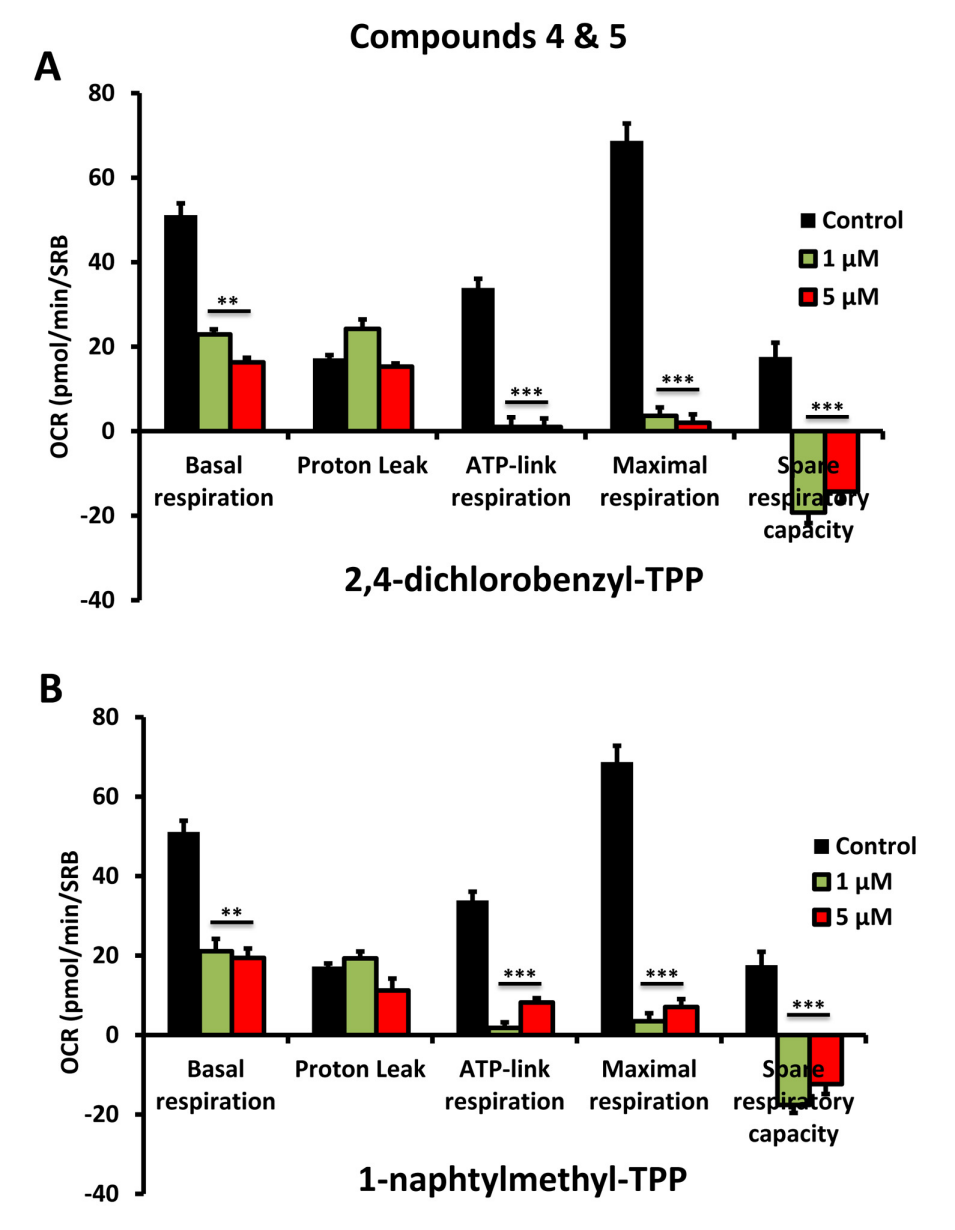
**Impaired mitochondrial function of MCF-7 cells after treatment with various TPP derivatives: Compounds 4 and 5.** Oxygen consumption rate (OCR) was measured with a Seahorse XF96 Extracellular Flux Analyzer. Data are represented as mean +/- SEM. Note that 2,4-dichlorobenzyl-TPP and 1-naphtylmethyl-TPP both effectively inhibit mitochondrial oxygen consumption. **p < 0.01; ***p < 0.001; indicates significance, all relative to the control.

To further validate that the effects we observed on reducing ATP levels were indeed due to the inhibition of mitochondrial function, we directly measured mitochondrial oxygen consumption rates (OCR) using the Seahorse XFe96 metabolic flux analyser. The results are shown in Figures 6-8. All five compounds behaved similarly, and effectively reduced basal mitochondrial respiration, with an IC-50 of approximately 1 μM.

**Figure 9:**
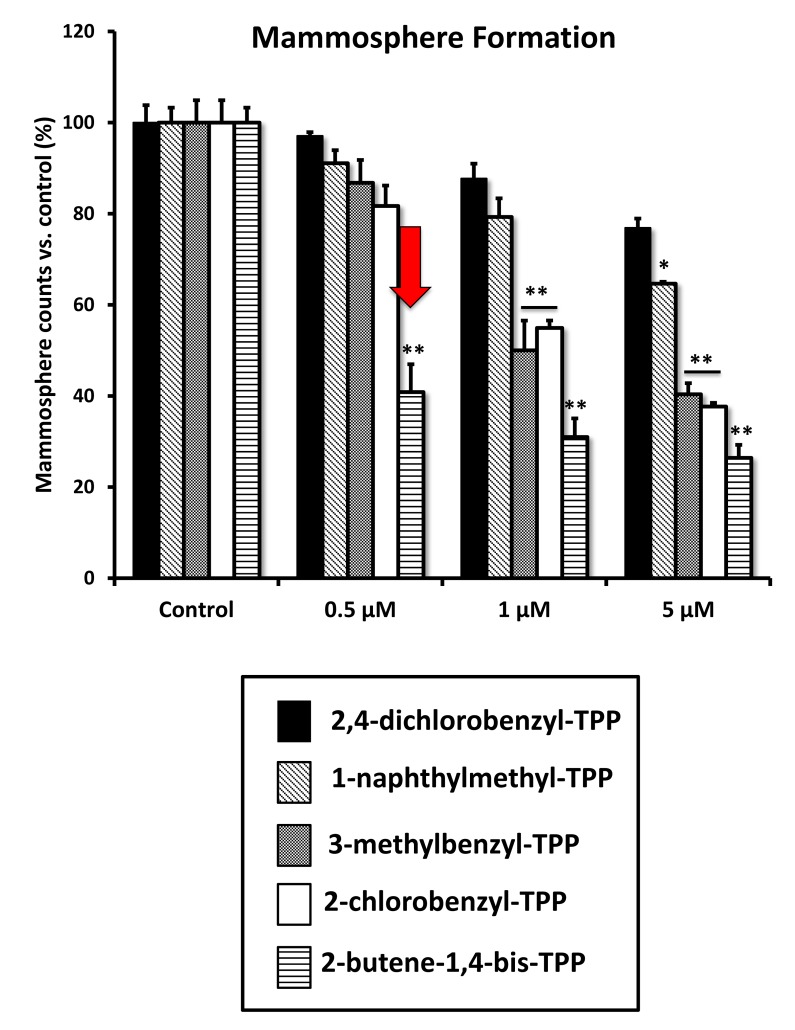
**Differential inhibition of the mammosphere-forming activity of MCF-7 breast CSCs, after treatment with various TPP derivatives.** 2,4-dichlorobenzyl-TPP (black); 1-naphthylmethyl-TPP (inclined lines); 3-methylbenzyl-TPP (dotted lines); 2-chlorobenzyl-TPP (white); 2-butene-1,4-bis-TPP (horizontal lines). Cells were treated for 5 days in mammosphere media. Data are represented as mean +/- SEM. Note that 2-butene-1,4-bis-TPP was the most effective compound for blocking CSC propagation, with an IC-50 less than 500 nM. *p < 0.05; **p < 0.01; indicates significance, all relative to the control.

We next evaluated the effects of these TPP compounds on the propagation of CSCs, using the mammosphere assay as a read-out (Figure 9). Interestingly, 2-butene-1,4-bis-TPP was the most effective, with an IC-50 < 500 nM. In contrast, for two of the other compounds tested (2-chlorobenzyl-TPP; 3-methylbenzyl-TPP) the IC-50 was between 1 to 5 μM. Finally, 1-naphthylmethyl-TPP was the least potent, with an IC-50 > 5 μM. Therefore, we conclude that 2-butene-1,4-bis-TPP is 2- to 10-fold more potent than the other TPP compounds, for inhibiting CSC propagation. This is despite the fact that they all behaved nearly identically in reducing mitochondrial respiration and ATP production. Therefore, another intrinsic property of 2-butene-1,4-bis-TPP must allow it to better target CSCs.

**Figure 10:**
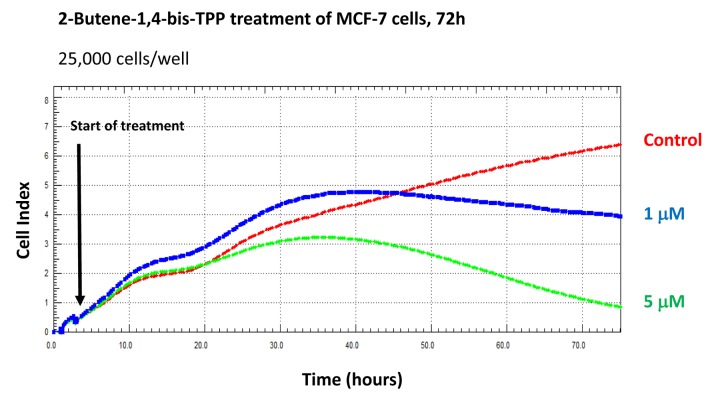
**Using the xCELLigence to assess the effects of 2-butene-1,4-bis-TPP on cell proliferation and viability/cell cycle arrest.** The xCELLigence system allows the real-time, label-free, monitoring of cell health and behavior, via high frequency measurement of cell-induced electrical impedance. Note that the effects of 2-butene-1,4-bis-TPP are concentration-dependent and most notable after 72 hours, but are also noticeable at 48 hours. Little or no effect was observed after 24 hours of incubation. Interestingly, 2-butene-1,4-bis-TPP was cytostatic at 1 μM.

Finally, we used the xCELLigence system to visualize the kinetics of the effectiveness of 2-butene-1,4-bis-TPP on the proliferation of adherent MCF-7 cells, for further validation. The xCELLigence system allows the real-time, label-free, monitoring of cell health and behavior, via high frequency measurement of cell-induced electrical impedance. Figure 10 shows that the effects of 2-butene-1,4-bis-TPP are concentration-dependent and most notable after 72 hours, but are also noticeable at 48 hours. Interestingly, 2-butene-1,4-bis-TPP was cytostatic at 1 μM. However, little or no effect was observed after 24 hours of incubation, implying that the compound is not immediately cytotoxic.

## DISCUSSION

**Figure 11:**
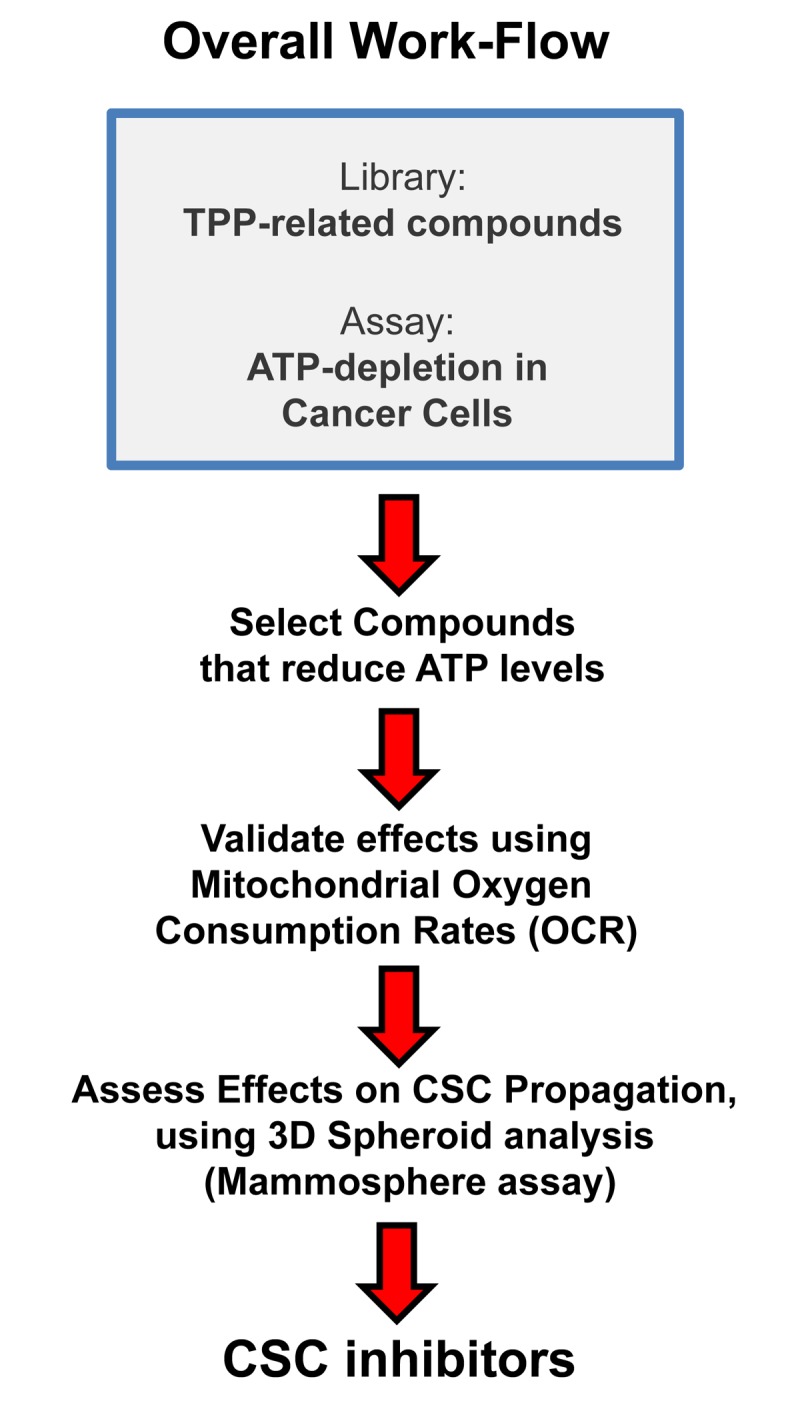
**Overall Work-Flow: Identifying mitochondrial inhibitors to target CSC propagation.** Here, we selected TPP-related compounds as the starting point for screening, because this ensures that all the compounds tested are targeted to mitochondria. Remarkably, we show that this strategy yields mitochondrial inhibitors of CSCs that are non-toxic in normal human fibroblasts, thereby effectively limiting drug toxicity.

**Figure 12:**
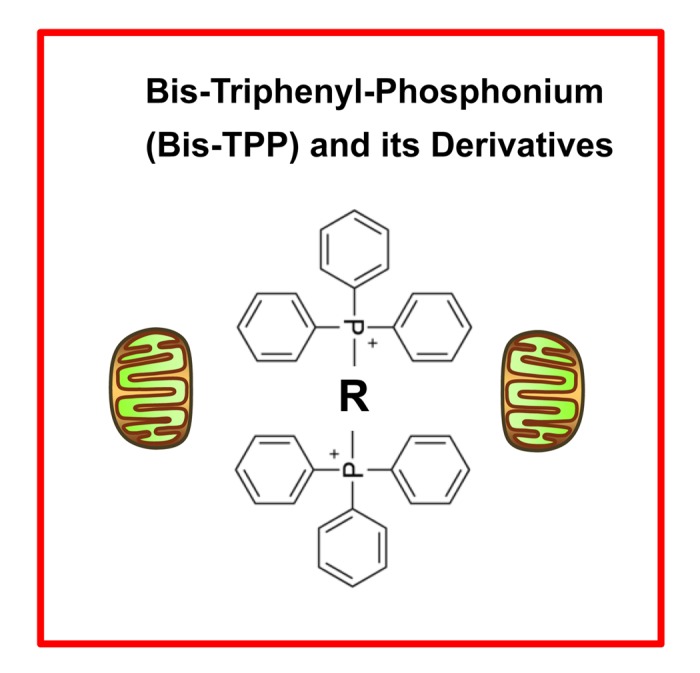
**bis-TPP: A mitochondrial targeting signal (MTS) for eradicating cancer stem cells (CSCs).** The “dimeric” structure of bis-TPP is shown, where R represents any chemical group or moiety.

TPP is a mitochondrial targeting signal, which is non-toxic in normal cells [21]. We hypothesized that TPP-tagged molecules could be employed to inhibit mitochondrial function in CSCs (Figure 11). To test this hypothesis, here we used an ATP depletion assay to screen the activity of nine randomly selected TPP compounds. As a consequence, we found five TPP-related compounds that significantly suppressed ATP levels, which yields a hit-rate of more than 50%. All five positive hit compounds were subjected to functional validation with the Seahorse XFe96, to quantitate their effects on the mitochondrial oxygen consumption rate (OCR). Remarkably, these TPP hit compounds were non-toxic in normal human fibroblasts and did not affect their viability or ATP production, showing striking selectively for cancer cells. Most importantly, these TPP hit compounds successfully blocked CSC propagation, as shown by employing the 3D spheroid assay. For example, 2-butene-1,4-bis-TPP was the most potent molecule that we identified, which targeted CSC propagation with an IC-50 < 500 nM. Interestingly, 2-butene-1,4-bis-TPP contains two TPP groups. This suggests that the use of a bis-TPP moiety may function as a “dimeric” or “polymeric” signal for the more effective targeting of mitochondria in CSCs (Figure 12). However, further studies will be necessary to fully explore the potential of bis-TPP.

## CONCLUSIONS

**Figure 13:**
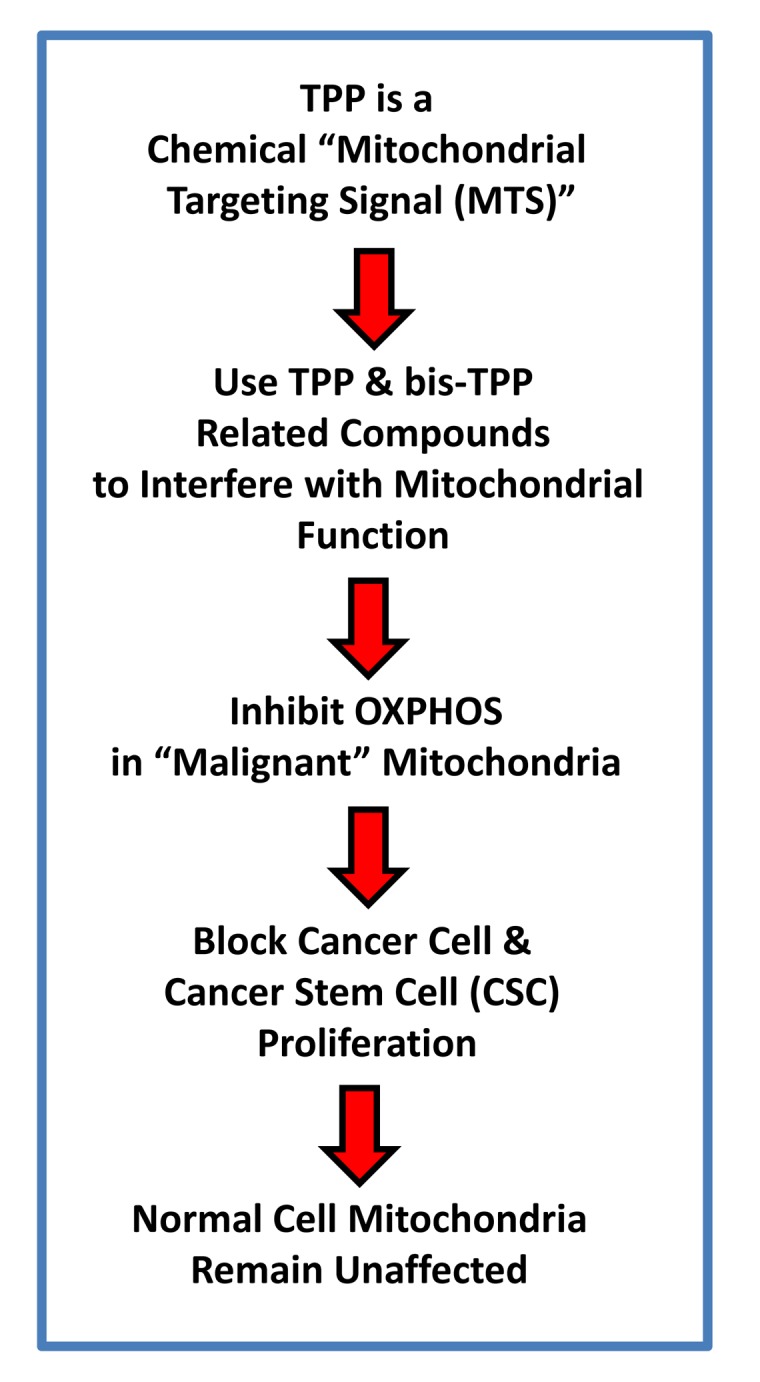
**TPP-related compounds provide a novel chemical strategy for targeting “bulk” cancer cells and CSCs, while minimizing off-target side-effects in normal cells.** Our results suggest that “malignant” mitochondria are functionally distinct from “normal” mitochondria. See text for further details.

**Figure 14:**
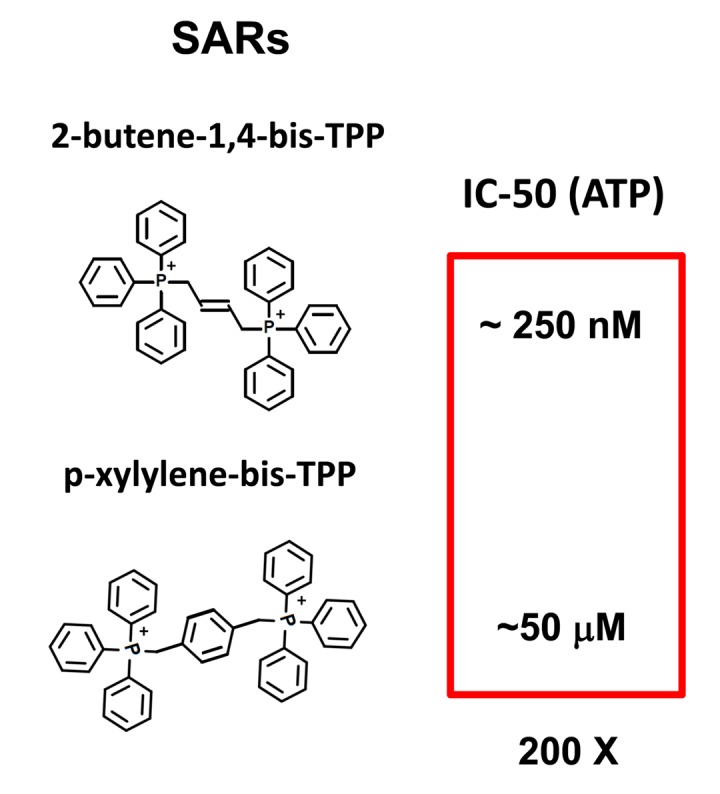
**bis-TPP: Structural activity relationships (SARs).** Comparison of 2-butene-1,4-bis-TPP (see Figures 1 and 2) with p-xylylene-bis-TPP (see Figure 1 and Table 1), the latter of which is approximately 200-fold less potent, in the context of ATP depletion.

In summary, we conclude that TPP-related compounds may provide a novel chemical strategy for effectively targeting “bulk” cancer cells and CSCs, while minimizing off-target side-effects in normal cells (Figure 13). In this context, bis-TPP may represent a more potent and selective form of TPP, especially for targeting CSCs. However, part of this potency and selectivity may also come from the reactive double bond in the central butene moiety, as p-xylylene-bis-TPP (Table 1) was ~200 times less effective than 2-butene-1,4-bis-TPP (Figure 2), in reducing overall ATP levels. See Figure 14 for a side-by-side structural comparison of these two related molecules.

## MATERIALS AND METHODS

### Cell culture and reagents

The human breast adenocarcinoma cell line (MCF-7) was purchased from the American Type Culture Collection (ATCC). hTERT-BJ1 cells were originally purchased from Clontech, Inc. MCF-7 and hTERT-BJ1 cells were grown in DMEM supplemented with 10% fetal bovine serum, GlutaMAX and 1% penicillin-streptomycin and incubated at 37C in a humidified 5% CO2 incubator. The medium was changed 2-3 times/week. Nine random TPP derivatives were purchased from Santa Cruz Biotechnology, Inc., as follows: (1) 2-butene-1,4-bis-TPP; (2) 2-chlorobenzyl-TPP; (3) 3-methylbenzyl-TPP; (4) 2,4-dichlorobenzyl-TPP; (5) 1-naphthylmethyl-TPP; (6) mito-TEMPO; (7) cyanomethyl-TPP; (8) p-xylylene-bis-TPP; (9) 4-cyanobenzyl-TPP.

### ATP-depletion assay (with CellTiter-Glo & Hoechst 33342)

MCF-7 cells were treated with different TPP derivatives for 72 hours in a black 96-well plate then wells were washed with PBS and were stained with Hoechst 33342 dye at a final concentration of 10 μg/ml. Fluorescence was read by a plate reader at 355 nm (excitation), 460 nm (emission). After washing with PBS CellTiterGlo luminescent assay (Promega) was performed according to the manufacturer’s protocols to determine intracellular ATP levels in the Hoechst dye stained cells. Both the fluorescent and luminescent data were normalized to control levels and were shown as percentage for comparison.

### Measuring the mitochondrial oxygen consumption rate (OCR)

Mitochondrial function was determined by using the XF Cell Mito Stress Test Kit (Seahorse Bioscience, MA, USA) with a Seahorse XFe96 Extracellular Flux Analyzer (Seahorse Bioscience, MA, USA). MCF-7 cells were seeded in a specialized 96-well tissue culture plate (XF96 microplate). Next day TPP derivatives were added and the plate was incubated for 72 hours. Before the experiment media was changed to XF base medium (including 1 mM pyruvate, 2 mM glutamine and 10 mM glucose) and cells were incubated at 37°C in a CO_2_-free atmosphere for one hour before measurement. After detection of basal oxygen consumption rate (OCR) (an indicator for mitochondrial respiration) OCR responses were evaluated towards the application of oligomycin (1 µM), FCCP (600 nM), and the combination of antimycin (1 µM) and rotenone (1 µM). From these measurements various parameters of mitochondrial function were determined. To determine cell viability in the measured wells sulphorodamine (SRB) assay was performed. Oxygen consumption rate (OCR) values were then normalized to the given SRB values.

### 3D Spheroid (mammosphere) assay

A single cell suspension of MCF-7 cells was prepared using enzymatic (1x Trypsin-EDTA, Sigma Aldrich, #T3924) and manual disaggregation (25 gauge needle) to create a single cell suspension. Cells were plated at a density of 500 cells/cm^2^ in mammosphere medium (DMEM-F12 media including B27/20 ng/ml and EGF/PenStrep) in non-adherent conditions, in culture dishes coated with (2-hydroxyethylmethacrylate) (poly-HEMA, Sigma, #P3932). Different TPP derivatives were previously diluted in the mammosphere media before addition of cells. Plates were maintained in a humidified incubator at 37°C at an atmospheric pressure in 5% (v/v) carbon dioxide/air. After 5 days of culture, spheres >50 μm were counted using an eyepiece graticule and mammosphere numbers were normalized to control treatments (cells treated with vehicle only).

### xCELLigence RTCA System (ACEA Biosciences Inc.)

Briefly, 25,000 MCF-7 cells were seeded in each well and used to assess the efficacy of 2-butene-1,4-bis-TPP, via RTCA (real-time cell analysis), via the measurement of cell-induced electrical impedance. This approach allows the quantification of the onset and kinetics of the cellular response.

## References

[1] Martinez-Outschoorn UE, Peiris-Pagés M, Pestell RG, Sotgia F, Lisanti MP. Cancer metabolism: a therapeutic perspective. Nat Rev Clin Oncol. 2017; 14:11–31. 10.1038/nrclinonc.2016.60 27141887

[2] Peiris-Pagès M, Martinez-Outschoorn UE, Pestell RG, Sotgia F, Lisanti MP. Cancer stem cell metabolism. Breast Cancer Res. 2016; 18:55. 10.1186/s13058-016-0712-6 27220421 PMC4879746

[3] Scopelliti A, Cammareri P, Catalano V, Saladino V, Todaro M, Stassi G. Therapeutic implications of Cancer Initiating Cells. Expert Opin Biol Ther. 2009; 9:1005–16. 10.1517/14712590903066687 19545218

[4] Duggal R, Minev B, Geissinger U, Wang H, Chen NG, Koka PS, Szalay AA. Biotherapeutic approaches to target cancer stem cells. J Stem Cells. 2013; 8:135–49. 24699023

[5] Zhang M, Rosen JM. Stem cells in the etiology and treatment of cancer. Curr Opin Genet Dev. 2006; 16:60–64. 10.1016/j.gde.2005.12.008 16377171

[6] Chandler JM, Lagasse E. Cancerous stem cells: deviant stem cells with cancer-causing misbehavior. Stem Cell Res Ther. 2010; 1:13. 10.1186/scrt13 20587011 PMC2905089

[7] Brooks MD, Burness ML, Wicha MS. Therapeutic Implications of Cellular Heterogeneity and Plasticity in Breast Cancer. Cell Stem Cell. 2015; 17:260–71. 10.1016/j.stem.2015.08.014 26340526 PMC4560840

[8] Lamb R, Harrison H, Hulit J, Smith DL, Lisanti MP, Sotgia F. Mitochondria as new therapeutic targets for eradicating cancer stem cells: quantitative proteomics and functional validation via MCT1/2 inhibition. Oncotarget. 2014; 5:11029–37. 10.18632/oncotarget.2789 25415228 PMC4294326

[9] Lamb R, Bonuccelli G, Ozsvári B, Peiris-Pagès M, Fiorillo M, Smith DL, Bevilacqua G, Mazzanti CM, McDonnell LA, Naccarato AG, Chiu M, Wynne L, Martinez-Outschoorn UE, et al. Mitochondrial mass, a new metabolic biomarker for stem-like cancer cells: understanding WNT/FGF-driven anabolic signaling. Oncotarget. 2015; 6:30453–71. 10.18632/oncotarget.5852 26421711 PMC4741544

[10] Farnie G, Sotgia F, Lisanti MP. High mitochondrial mass identifies a sub-population of stem-like cancer cells that are chemo-resistant. Oncotarget. 2015; 6:30472–86. 10.18632/oncotarget.5401 26421710 PMC4741545

[11] Lamb R, Ozsvari B, Lisanti CL, Tanowitz HB, Howell A, Martinez-Outschoorn UE, Sotgia F, Lisanti MP. Antibiotics that target mitochondria effectively eradicate cancer stem cells, across multiple tumor types: treating cancer like an infectious disease. Oncotarget. 2015; 6:4569–84. 10.18632/oncotarget.3174 25625193 PMC4467100

[12] Bonuccelli G, De Francesco EM, de Boer R, Tanowitz HB, Lisanti MP. NADH autofluorescence, a new metabolic biomarker for cancer stem cells: identification of Vitamin C and CAPE as natural products targeting “stemness”. Oncotarget. 2017; 8:20667–78. 10.18632/oncotarget.15400 28223550 PMC5400535

[13] Bonuccelli G, Peiris-Pages M, Ozsvari B, Martinez-Outschoorn UE, Sotgia F, Lisanti MP. Targeting cancer stem cell propagation with palbociclib, a CDK4/6 inhibitor: telomerase drives tumor cell heterogeneity. Oncotarget. 2017; 8:9868–84. 10.18632/oncotarget.14196 28039467 PMC5354777

[14] De Luca A, Fiorillo M, Peiris-Pagès M, Ozsvari B, Smith DL, Sanchez-Alvarez R, Martinez-Outschoorn UE, Cappello AR, Pezzi V, Lisanti MP, Sotgia F. Mitochondrial biogenesis is required for the anchorage-independent survival and propagation of stem-like cancer cells. Oncotarget. 2015; 6:14777–95. 10.18632/oncotarget.4401 26087310 PMC4558115

[15] Fiorillo M, Sotgia F, Sisci D, Cappello AR, Lisanti MP. Mitochondrial “power” drives tamoxifen resistance: NQO1 and GCLC are new therapeutic targets in breast cancer. Oncotarget. 2017; 8:20309–27. 10.18632/oncotarget.15852 28411284 PMC5386764

[16] Fiorillo M, Lamb R, Tanowitz HB, Cappello AR, Martinez-Outschoorn UE, Sotgia F, Lisanti MP. Bedaquiline, an FDA-approved antibiotic, inhibits mitochondrial function and potently blocks the proliferative expansion of stem-like cancer cells (CSCs). Aging (Albany NY). 2016; 8:1593–607. 10.18632/aging.100983 27344270 PMC5032685

[17] Fiorillo M, Lamb R, Tanowitz HB, Mutti L, Krstic-Demonacos M, Cappello AR, Martinez-Outschoorn UE, Sotgia F, Lisanti MP. Repurposing atovaquone: targeting mitochondrial complex III and OXPHOS to eradicate cancer stem cells. Oncotarget. 2016; 7:34084–99. 10.18632/oncotarget.9122 27136895 PMC5085139

[18] Lamb R, Fiorillo M, Chadwick A, Ozsvari B, Reeves KJ, Smith DL, Clarke RB, Howell SJ, Cappello AR, Martinez-Outschoorn UE, Peiris-Pagès M, Sotgia F, Lisanti MP. Doxycycline down-regulates DNA-PK and radiosensitizes tumor initiating cells: implications for more effective radiation therapy. Oncotarget. 2015; 6:14005–25. 10.18632/oncotarget.4159 26087309 PMC4546447

[19] Ozsvari B, Sotgia F, Lisanti MP. A new mutation-independent approach to cancer therapy: inhibiting oncogenic RAS and MYC, by targeting mitochondrial biogenesis. Aging (Albany NY). 2017; 9:2098–116. 10.18632/aging.101304 29080556 PMC5680558

[20] De Francesco EM, Maggiolini M, Tanowitz HB, Sotgia F, Lisanti MP. Targeting hypoxic cancer stem cells (CSCs) with Doxycycline: implications for optimizing anti-angiogenic therapy. Oncotarget. 2017; 8:56126–42. 10.18632/oncotarget.18445 28915578 PMC5593549

[21] Ross MF, Prime TA, Abakumova I, James AM, Porteous CM, Smith RA, Murphy MP. Rapid and extensive uptake and activation of hydrophobic triphenylphosphonium cations within cells. Biochem J. 2008; 411:633–45. 10.1042/BJ20080063 18294140

